# Automatic Annotation of Retinal Layers in Optical Coherence Tomography Images

**DOI:** 10.1007/s10916-019-1452-9

**Published:** 2019-11-13

**Authors:** Bashir Isa Dodo, Yongmin Li, Khalid Eltayef, Xiaohui Liu

**Affiliations:** Department of Computer Science, Brunel University London, Kingston Lane, Uxbridge, UB83PH UK

**Keywords:** Retinal layer segmentation, Optical coherence tomography, Graph-Cut, Speckle noise

## Abstract

Early diagnosis of retinal OCT images has been shown to curtail blindness and visual impairments. However, the advancement of ophthalmic imaging technologies produces an ever-growing scale of retina images, both in volume and variety, which overwhelms the ophthalmologist ability to segment these images. While many automated methods exist, speckle noise and intensity inhomogeneity negatively impacts the performance of these methods. We present a comprehensive and fully automatic method for annotation of retinal layers in OCT images comprising of fuzzy histogram hyperbolisation (FHH) and graph cut methods to segment 7 retinal layers across 8 boundaries. The FHH handles speckle noise and inhomogeneity in the preprocessing step. Then the normalised vertical image gradient, and it’s inverse to represent image intensity in calculating two adjacency matrices and then the FHH reassigns the edge-weights to make edges along retinal boundaries have a low cost, and graph cut method identifies the shortest-paths (layer boundaries). The method is evaluated on 150 B-Scan images, 50 each from the temporal, foveal and nasal regions were used in our study. Promising experimental results have been achieved with high tolerance and adaptability to contour variance and pathological inconsistency of the retinal layers in all (temporal, foveal and nasal) regions. The method also achieves high accuracy, sensitivity, and Dice score of 0.98360, 0.9692 and 0.9712, respectively in segmenting the retinal nerve fibre layer. The annotation can facilitate eye examination by providing accurate results. The integration of the vertical gradients into the graph cut framework, which captures the unique characteristics of retinal structures, is particularly useful in finding the actual minimum paths across multiple retinal layer boundaries. Prior knowledge plays an integral role in image segmentation.

## Introduction

Ophthalmic imaging technologies has witnessed an ever-growing scale of retina images, both in volume and variety. Nowadays the 2D fundus images are widely available in the high-street opticians, while the recent 3D Optical Coherence Tomography (OCT) [17] images have gradually become a common imaging modality in clinical practice. However, this vast amount of imaging data are largely stored in their raw format. Even after diagnosis and treatment, the relevant medical information provided by the clinical experts, if any, is normally recorded separately from the images. Apparently the lack of high-level information on the retinal image, e.g. labels, tags, markers and measures, has hindered the development of new methods of diagnosis and treatment. To a greater level, this has also presented a significant challenge to healthcare analytics.

Motivated by the aforementioned challenge, in this work we aim to develop a comprehensive and fully automatic method for annotation of retinal layers in OCT images. This will provide the most basic but yet important structural information to the original raw data, and serve as a starting step for any further and large-scale healthcare analytics.

**Fig. 1:**
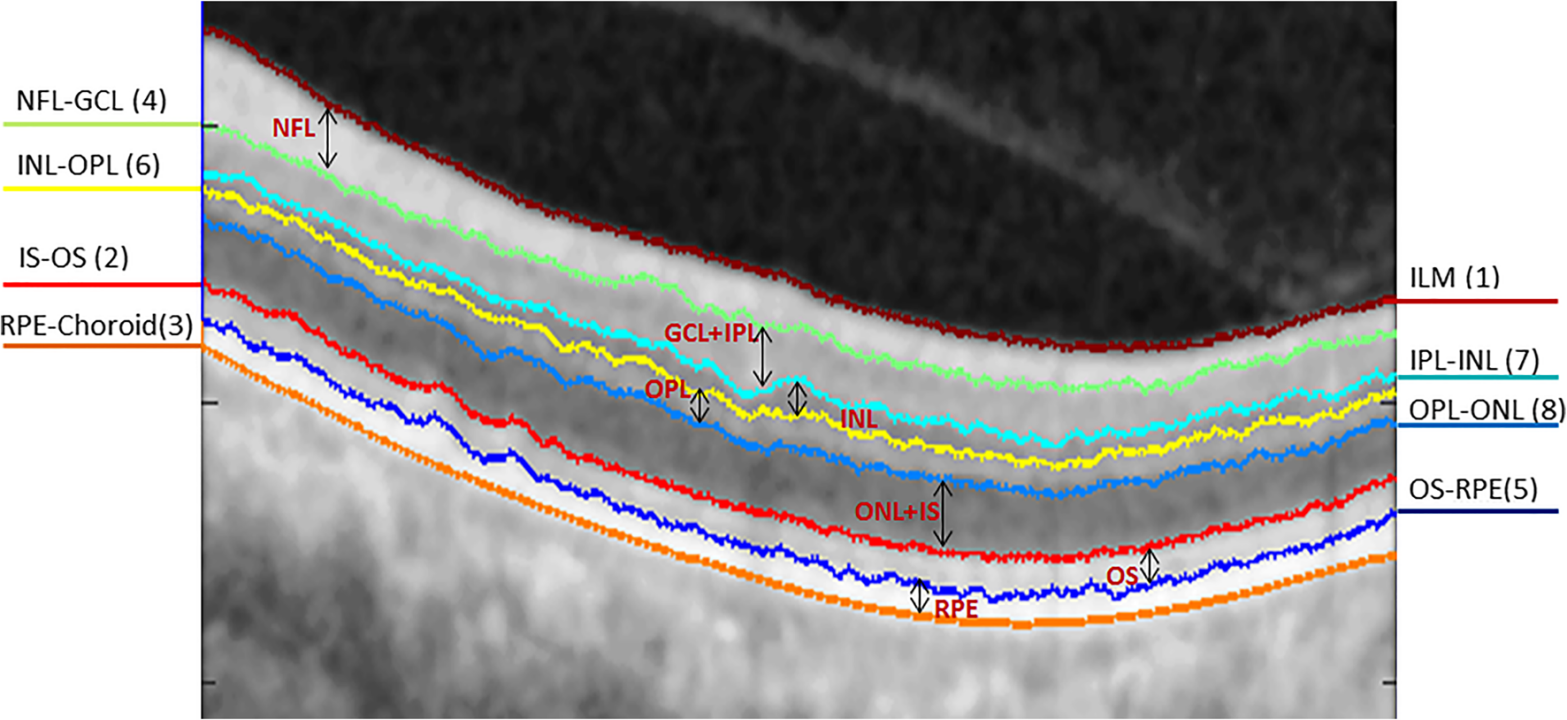
Illustration of the 8 boundaries and 7 retinal layers segmented in the study. The numbers in brackets are the sequential order of the segmentation

In particular, we take into account the effect of promoting continuity and discontinuity to improve segmentation accuracy. In addition, we impose hard constraints based on the structure of retina to segment seven retinal layers including the Nerve Fibre Layer (NFL), the Ganglion Cell to Layer-Inner Plexiform Layer (GCL+IPL), the Inner Nuclear Layer (INL), the Outer Plexiform Layer (OPL), the Outer Nuclear Layer to Inner Segment (ONL+IS), the Outer Segment (OS) and the Retinal Pigment Epithelium (RPE) by detecting eight layer boundaries. The locations of these layers and boundaries are illustrated in Fig. 1.

This paper is organized as follows. In Section “Background”, we review previous work on noise handling and retinal layer segmentation of OCT images. Section “Retinal layer segmentation method” describes the proposed segmentation method. Section “Experimental results” presents experimental results on 150 OCT images and discussion. Finally conclusions are drawn in Section “Conclusions”.

## Background

### Optical coherence tomography

OCT imaging [17] has become one of the best tools for diagnosis of retinal diseases [21]. Time-Domain OCT (TD-OCT) is one of the first modalities used in retinal diagnosis, however due to it’s shortcomings the Spectral-Domain OCT (SD-OCT) [11, 49] was introduced to handle these limitations. Since its introduction various protocols and modalities based on TD-OCT, SD-OCT, FD-OCT and different machines such as Stratus, Spectralis, Cirrus, Optovue etc and their various versions provides variety of information regarding retinal layer anatomy. The segmentation of various retinal layers from OCT is vital for tracking progress of medication and diagnosing various ocular diseases, in particular for Diabetic Retinopathy, Glaucoma and Age Related Macula Degeneration (AMD). This is especially intriguing as they are not noticed early enough by the patient, and usually cause irreversible impairment. Manual OCT segmentation is tedious, time-consuming, and suffers from inter- and intra-rater variability. Automated segmentation, on the other hand, holds the potential to reduce the time and effort required to delineate the retinal layers and also to provide repeatable, quantitative results. Additionally, considering the large number and variety of images which exist, it is not sustainable commercially to develop a new algorithm for each application [48].

### Noise and noise handling in retinal OCT

In OCT images, two main kinds of noise exists i.e the speckle noise during acquisition and the shadows of blood vessels. Speckle noise in OCT images causes difficulty in the precise identification of the boundaries of layers or other structural features in the image either through direct observation or use of segmentation algorithms [1, 28]. The noise that corrupts OCT images is non-Gaussian, multiplicative and neighborhood correlated. Thus, it is not easily suppressed by standard software denoising methods [27]. Since OCT images are highly corrupted by speckle noise, some pre-processing steps are usually performed to reduce the effect of noise. In most cases, even though the segmentation algorithms are designed to handle uncertainties and noise, the pre-processing is used as a first step to handling the noise, irrespective of whether the analysis to be performed is in 2D [4, 21, 42] or 3D [43, 45, 46], in order to remove the speckle noises and enhance the contrast between layers. Usually the de-noising is achieved by using the 3D anisotropic diffusion method, 3D median filter, 3D Gaussian filter or 3D wavelet transform [47]. Previous attempts including spatial and frequency compounding techniques have been used to address the problem of speckle noise in OCT [18, 30] . However, the tolerance or adaptability of these techniques are limited, which then complicates the analysis stage. They are also quite sensitive to the choice and fine tuning of various parameters [36, 37].

On the other hand, a number of digital filters have been used for speckle suppression on OCT images, such as median filtering, wavelet-based filtering that employs nonlinear thresholds, anisotropic diffusion filtering [12] , and nonlinear anisotropic filtering [15]. While most of these methods are effective in reducing speckle noise, some of them tend to blur the structural boundaries in the OCT image. As a matter of fact, most of these algorithms use a defined filter window to estimate the local noise variance of a speckle image and perform the individual unique filtering process. The result is generally a reduced speckle level in areas that are homogeneous. But the image is either blurred or over smoothed due to losses in detail in non-homogeneous areas like edges or lines. Also, the conventional algorithms in OCT segmentation do not consider the intensity inhomogeneity in the image which can lead to inaccurate segments and inability to detect all layers. Clearly, the primary goal of noise reduction is to remove the noise without losing much detail contained in an image [36]. We propose a method, that preserves the edge information, and improve the visibility by hyperbolizing the image. This improves homogeneity of pixel values in every layer, which consequently improves performance of the segmentation method, and makes our method applicable, for diagnosis and tracking medication progress of ocular diseases.

### Segmentation of retinal layers

The segmentation of retinal layers has been an area of active research and has drawn a large number of researches since the introduction of OCT. Various methods have been proposed, some with focus on the number of layers to be segmented, others on the computational efficiency. Segmentation of retinal images is challenging and requires automated analysis methods. In this regard a multi-step approach was developed by [2]. However the results obtained were highly dependent on the quality of images and the alterations induced by retinal pathologies. A 1-D edge detection algorithm using the Markov Boundary Model [23], which was later extended by [3] to obtain the optic nerve head and RNFL. Seven layers were obtained by [4] using a peak search interactive boundary detection algorithm based on local coherence information of the retinal structure. Statistical methods such as Expectation maximisatin and probabilistic modelling were reported in [19, 20]. The Level Set method was used by [29, 42, 44, 46] which were computationally expensive compared to other optimization methods. Graph based methods in [14, 16, 21, 31–35] have reported successful segmentation results, with varying success rates. Recently, a method using the Fuzzy Histogram Hyperbolization (FHH) is proposed to improve the image quality, and then to be embedded into the continuous max-flow to simultaneously segment four retinal layers [9].

Moreover, the use of gradient information derived from the retinal structures has in recent years been of interest to OCT segmentation researchers. It was utilised by [5] with the Graph-Cut method, where the retinal structure is employed to limit search space and reduced computational time with dynamic programming. This method was recently extended to 3D volumetric analysis by [38] in OCTRIMA 3D with edge map and convolution kernel in addition to hard constraints in calculating weights. They also exploited spatial dependency between adjacent frames to reduce processing time. Edge detection and polynomial fitting was yet another approach proposed to derive boundaries of the retinal layers from gradient information by [25], and machine learning by [24] with the use of random forest classifier. The utilization of gradient information on OCT images is largely based on the changes that occur at layer boundaries in the vertical direction, thereby attracting segmentation algorithms to exploit this advantage. The paths obtained by the default shortest path algorithms, have no optimal way of handling inconsistencies (such as the irregularity in OCT images), as thus it sometimes obtains the wrong paths, which we refer to as “wrong short-cuts”. To handle this issue, we reassign the weights to promote homogeneity between adjacent edges, such that transition at layer boundaries become clearer, and this contributes largely to the success of our graph-cut approach. Our method takes into account the retinal structure and gradient information, but more importantly, the re-assignment of weights in the adjacency matrix, because segmentation using graph cut methods, depends on assignment of appropriate weight.

## Retinal layer segmentation method

In this section we provide the details of our approach. We first enhance the images using the Fuzzy Histogram Hyperbolization (FHH) to enhance the image and suppress the noise, then segment 8 retinal layer boundaries from OCT B-Scan images. Parts of this work were presented in [7, 9]

### Enhancement

Every image *I*, is represented by the following [13]:

1 $$ \textit{I} = \bigcup\limits^{{M}}_{{m}}\bigcup\limits^{{N}}_{{n}} \frac{\mu_{mn}}{g_{mn}} $$

Where *g*_*mn*_ represents the intensity of the *mn*^*th*^ pixel and *μ*_*mn*_ its membership value, given m = 1,2,3…M and n = 1,2,3…N. In line with this, using the linear index of fuzziness, we calculated image fuzziness with [39] :

2 $$ \gamma(\textit{I}) = \frac{2}{MN} \sum\limits_{{i= 1}}^{{N}} \sum\limits_{{j=1}}^{{M}} \min[\mu_{I}(g_{ij}),\bar{\mu}_{I}(g_{ij}) ] $$

where *μ*_*I*_(*g*_*ij*_) is the membership function of greylevel *g*_*ij*_ and $\bar {\mu }_{I}(g_{ij})$ = 1 - *μ*_*I*_(*g*_*ij*_). This maps image greylevel intensities into a fuzzy plane using membership functions. The membership functions are modified for contrast enhancement, and the fuzzy plane is mapped back to image grey level intensities. The aim is to generate an image of higher contrast than the original image by giving larger values to the greylevels that are closer to the mean greylevel of the image than to those that are farther from the mean.

Using the concept of histogram and fuzzy histogram hyperbolization described in [40] and [41], we calculate membership value for each greylevel as:

3 $$ \mu({g}_{mn})= \frac{g_{mn} - g_{\min}}{g_{\max} - g_{\min}}  $$

where the maximum and minimum intensity values are represented by $g_{\max \limits }$ and $g_{\min \limits }$ respectively. Then *β* as a fuzzifier and the desired grey level value L, are used to calculate the new grey values of image using the following transformation [41] :

4 $$ g^{\prime}_{mn} =\left( \frac{L-1}{ e^{-1}-1}\right) *\left[e^{-U(g_{mn})^{\beta}}-1\right]  $$

**Fig. 2:**
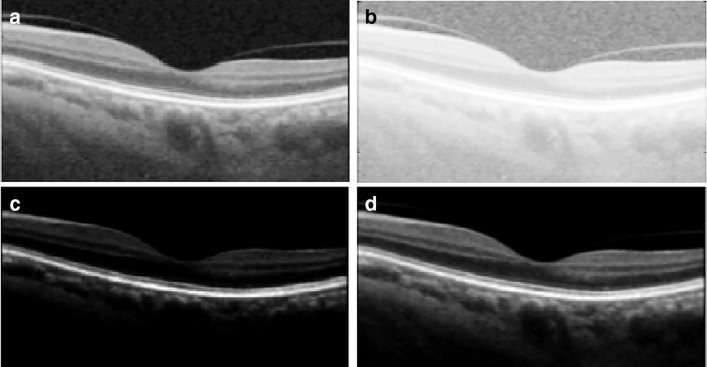
Image Enhancement. The unprocessed image is shown in A, and the transformed images with various values of *β*= 0.3 in B, *β* = 5 in C, and *β* = 2.2 in D (the computed value for this image)

Fuzzy histogram hyperbolization is simple and straight forward, yet effective to a range of image and signal processing applications [26]. The value of *β* determines a number of operations that could be performed with membership modification [40]. As the value of *β* approaches 0, the results are similar to that of histogram equalization, whereas if *β* approaches values 5 and above, it tends to provide result similar to segmentation. We therefore take two issues into consideration: 1) Most image de-noising processes are sensitive to the choice of various parameters [36]. 2) The fuzzifier *β* modifies the membership values additionally, and so, the gray level dynamics of the resulting image can be changed [40]. Consequently, the value of *β* from Eq. 4 is constrained to arbitrate within a specified window by the following conditions:

5 $$ \begin{array}{@{}rcl@{}} \beta &=& \beta + C\\ \beta &=& T_{\min} \qquad if \quad \beta + C < T_{\min}\\ \beta &=& T_{\max} \qquad if \quad \beta + C > T_{\max} \end{array} $$

where $T_{\min \limits }$ and $T_{\max \limits }$ are the minimum and maximum acceptable values of *β*. To achieve the above, we introduce constant *C*, called the stabilizer. The stabilizer keeps the value of *β* within the set threshold. This allows the method to set a suitable value for the image based on the membership information without the need for user input. Of course the threshold values can always be adjusted easily, for the method to adapt to a wider range of images and applications, however, we limit our study to the enhancement of retinal OCT images to suppress and handle speckle noise and blood vessel interference. After the transformation, the image is enhanced and this has positive effect in calculating the flow. Examples of image transformations with various values of *β* are shown in Fig. 2. For this image, *β* is set to 2.2 in our experiment and will vary depending on the image.

Unlike other preprocessing methods, which reduce image quality or leads to loss of data, this method preserves edge information and adapts to OCT inconsistencies as the value is computed based on each image. This allows the method to adapt to different OCT images.

### Weight calculation

**Fig. 3:**
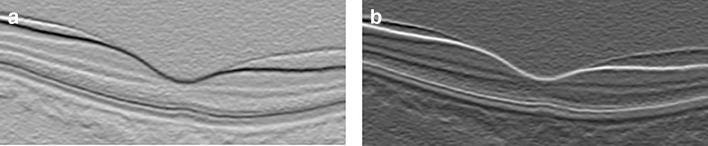
Image gradients used in generating **a** dark-bright adjacency matrix and **b** bright-dark adjacency matrix

In this stage we obtain the vertical gradient of the image, normalize the gradient image to values in the range of 0 to 1, and then we obtain the inverse of the normalized image gradient as shown in Fig. 3. These two normalized gradient images are then used to obtain two separate undirected adjacency matrices, where Fig. 3a and b contains information for transitions from bright-dark and dark-bright respectively. The adjacency matrices are formulated with the following equation [5]:

6 $$ w_{ab} = 2 - g_{a}-g_{b} +w_{\min} $$

where *w*_*ab*_, *g*_*a*_, *g*_*b*_ and $w_{\min \limits }$ are the weights assigned to the edge connecting any two adjacent nodes *a* and *b*, the vertical gradient of the image at node *a*, the vertical gradient of the image at node *b*, and the minimum weight added for system stabilization. To improve the continuity and homogeneity in the adjacency matrices they are hyperbolized, firstly by calculating the membership function with the fuzzy sets equation (3) [41] and then transformed with Eq. 8.

7 $$ w_{ab}^{\prime} = \frac{w_{ab} - w_{mn}}{w_{mx} - w_{mn}}  $$

where *w*_*mn*_ and *w*_*mx*_ represents the maximum and minimum values of the adjacency matrix respectively, the adjacency matrices are then transformed with the following equation:

8 $$ w_{ab}^{\prime\prime} = {(w_{ab}^{\prime})^{\beta_{w}}}  $$

where $w_{ab}^{\prime }$ is the membership value from Eq. 7, and *β*_*w*_, the fuzzifier is a constant. Considering the number of edges in an adjacency matrix, we use a constant *β*_*w*_ instead of calculating the fuzziness. The main reason is to reduce computational time and memory usage. The resulting adjacency matrices are such that the weights are reassigned, and the edges with high weights get higher values while those with low values get lower edge weights. Our motive here is that, if continuity or discontinuity is re-emphasized the algorithm will perform better, where in this case we improve both. The edges connecting pixels of the same region get higher values close to each other, while those connecting pixels of the background and layer boundaries gets lower along the way. This is more realistic and applicable in this context (as the shortest path is greedy search approach), because at the boundary of each layer there is a transition from bright to dark or dark to bright, and therefore improving it aids the algorithm in finding correct optimal solutions that are very close to the actual features of interest.

The weight calculation is followed by several sequential steps of segmentation that are discussed in the next few subsections. We adopt layer initialization from [5], where two columns are added to either side of the image with minimum weights ($w_{\min \limits }$), to enable the cut move freely in those columns. This is based on the understanding that each layer extends from the first to last column of the image, i.e. dividing the image horizontally at each layer boundary, and that the Graph-Cut method prefers paths with minimum weights. We use Djikstra’s algorithm [6] in finding the minimum weighted path in the adjacency matrix (other optimization methods utilizing sparse adjacency matrices might be used in finding the minimum path). Graph-Cut methods are optimal at finding one boundary at a time, and therefore to segment multiple regions in most cases, requires an iterative search in limited space. Limiting the region of search is a complex task, it requires prior knowledge and is dependent on the structure of the features or regions of interest. Some additional information on automatic layer initialization and region limitation are discussed in [5, 7, 21].

### ILM, IS-OS, RPE and NFL-GCL segmentation

It is commonly accepted that the NFL, IS-OS and RPE exhibits high reflectivity in an OCT image [5, 25, 38]. This is also evident from our previous work [9], where we segmented the four most reflective layers. Taking into account this reflectivity and the dark-bright transition we segment the ILM and IS-OS boundaries using Dijkstra’s algorithm [6]. The ILM (vitreous-NFL) boundary is segmented by searching for the highest change from dark-bright, this is because there is a sharp change in the transition, additionally it is amidst extraneous features, above it is the background region in addition to no interruption of the blood vessels, as can be seen in the gradient image. All of the above reasons make it easier to segment the ILM than other layers. We then limit the region below ILM and search for the next highest change from dark-bright in order to segment the IS-OS boundary. In most cases the ILM is segmented, but to account for uncertainties, i.e to differentiate or confirm which layer was segmented, we use the mean value of the vertical axis of the paths to determine the layer segmented, as the ILM is above the IS-OS (similar to [5].

As mentioned earlier, RPE is one of the most reflective layers. The RPE-Choroid boundary exhibits the highest bright-dark layer transition as can be seen in Fig. 3a. Additionally based on experimental results, it is better to search for the transition from bright to dark for the RPE, due to the interference of blood vessels and the disruption of hyper-reflective pixels in the choroid region. Therefore searching for the bright-dark transition is ideal for the RPE most especially to adapt to noisy images. To segment the NFL-GCL boundary,we limit the search space between ILM to IS-OS, and utilize the bright-dark adjacency matrix to find the minimum weighted path. The resulting path is the NFL-GCL boundary, as it is one of the most hyper-reflective layers. Additionally if we limit our search space to regions below the ILM and above the RPE, the resulting bright-dark and dark-bright minimum paths are the NFL-GCL and IS-OS respectively (i.e the NFL-GCL and IS-OS boundaries exhibits the second highest bright-dark and dark-bright transition respectively in an OCT image).

### OS and IPL to ONL segmentation

To segment the OS-RPE and three other boundaries (IPL-INL, INL-OPL, and OPL-ONL) from IPL to ONL, we use the prior segmented layers as benchmarks for search space limitation. We obtain the OS-RPE region by searching for the dark-bright shortest path between IS-OS and the RPE-Choroid. For the remaining boundaries, first we segment the INL-OPL, because it exhibits a different transition among the three. This is done by searching for the shortest path between NFL-GCL and IS-OS on the dark-bright adjacency matrix. Consequently the IPL-INL and OPL-ONL boundaries are obtained by limiting the region of path search between INL-OPL and NFL-GCL, and INL-OPL and IS-OS regions respectively, on the bright-dark adjacency matrix.

## Experimental results

We evaluated the performance of the proposed method on a set of 150 B-scan OCT images centred on the macular region. This data set was collected in Tongren Hospital with a standard imaging protocol for retinal diseases such as glaucoma. The resolution of the B-scan images are 512 pixels in depth and 992 pixels across section with 16 bits per pixel. We manually labelled all the retinal layers in the dataset under the supervision of clinical experts. This serves as the ground truth in our experiments. Prior to segmenting the images, 15% percent of the image height was cropped from the top to remove regions with low signal and no features of interest. We segment seven retinal layers automatically using MATLAB 2016a software. The average computation time was 4.25 seconds per image on a PC with Intel i5-4590 CPU, clock of 3.3GHz, and 8GB RAM memory.

**Fig. 4:**
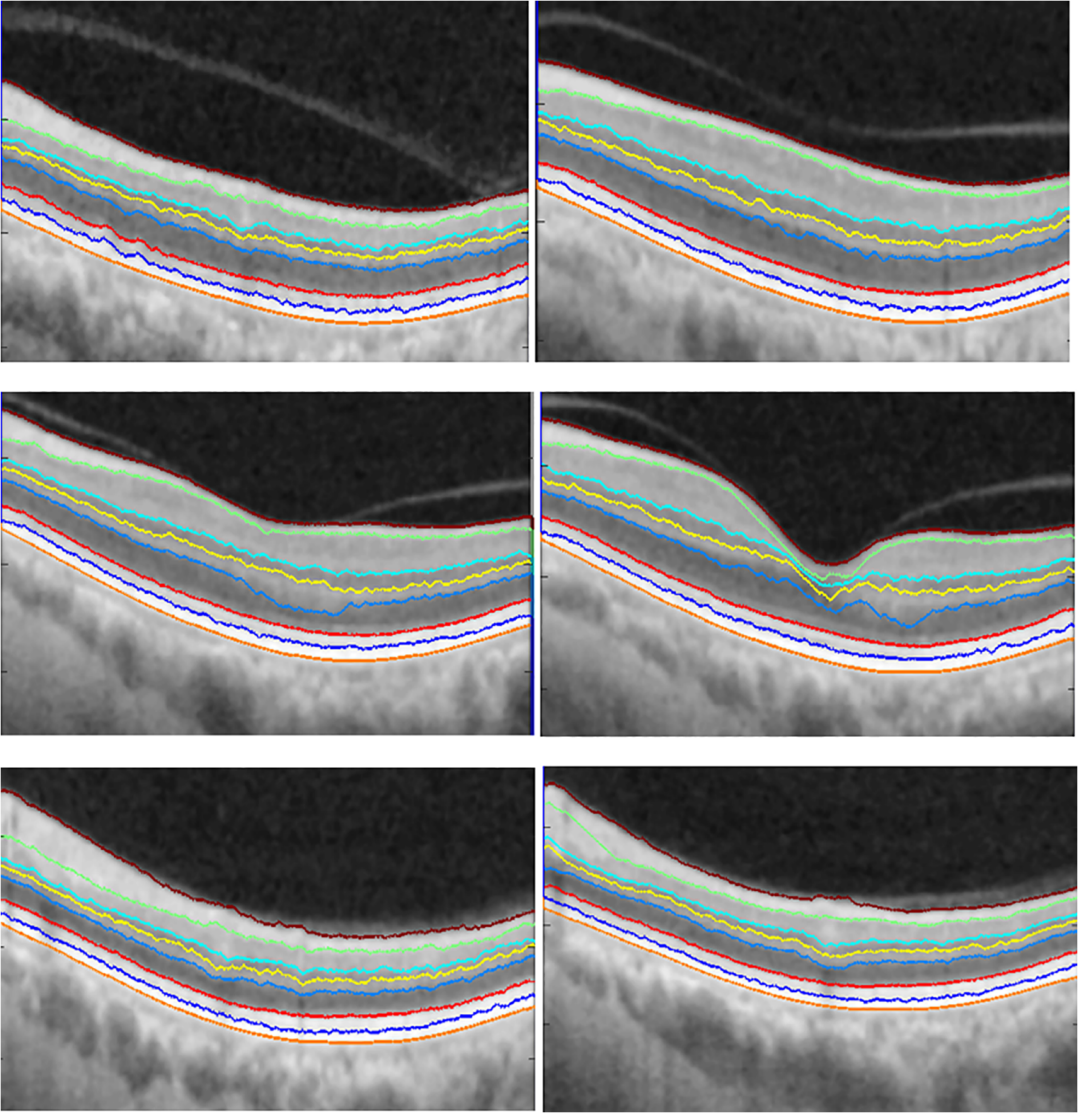
Segmentation results of 8 boundaries and 7 layers. Boundaries from top to bottom, the segmented boundaries are ILM, NFL-GCL, IPL-INL,INL-OPL, OPL-ONL, IS-OS, OS-RPE and RPE-Choroid

The method obtains the boundaries in the order from ILM(Vitreous-NFL), IS-OS, RPE-Choroid, NFL-GCL, OS-RPE, INL-OPL, IPL-INL to OPL-ONL respectively. The locations of these boundaries and the sequential order of the segmentation is shown in Fig. 1. Sample results of the 8 retinal layer boundaries and the underlying 7 layers are shown in Fig. 4. To evaluate the proposed method we calculate the Root Mean Squared Error (RMSE), and Mean Absolute Deviation (MAD) by Eq. 9. Table 1 shows the mean and standard deviation of both MAD and RMSE, for the seven layers targeted in this study.

9 $$ \begin{array}{@{}rcl@{}} MAD(GT,SEG) & = & 0.5 * \left( \frac{1}{n}\! \sum\limits_{i=1}^{n}d(pt_{i}, SEG) + \frac{1}{m} \!\sum\limits_{{i=1}}^{m}d(ps_{i}, GT) \!\right)\\ RMSE & = & \sqrt{\frac{1}{n} \sum\limits_{i=1}^{n}(SEG_{i} - GT_{i})^{2}}\\ Dice& = & \frac{2\mid GT_{i}\cap SEG_{i}\mid}{\mid GT_{i}\mid + \mid SEG_{i}\mid} \end{array} $$

where *SEG*_*i*_ is the pixel labelled as retinal Layer by the proposed segmentation method and *GT*_*i*_ is the true retinal layers pixel in the manually annotated image (ground truth) image. *pt*_*i*_ and *ps*_*i*_ represent the coordinates of the images, while *d*(*pt*_*i*_,*SEG*) is the distance of *pt*_*i*_ to the closest pixel on *SEG* with the same segmentation label, and *d*(*ps*_*i*_,*GT*) is the distance of *ps*_*i*_ to the closest pixel on *GT* with the same segmentation label. *n* and *m* are the number of points on *SEG* and *GT* respectively. For all layers our method has performed well. Especially considering the low value of NFL for both MAD and RMSE. The high value in ONL+IS is due to the presence of high noise and lower reflectivity of the boundaries within the region, however, this is still considerably low.

Furthermore, We evaluated the retinal nerve fibre layer thickness (RNFLT) (the area between ILM and NFL-GCL) with additional criteria, due to its high importance in the diagnosis of ocular diseases, including glaucoma. This is evaluated with four criteria, namely, accuracy, sensitivity(true positive rate(TPR)), error rate(FPR) and the Dice index(coefficient). These measurements are computed with the following equations while the Dice is computed from Eq. 10:

10 $$ \begin{array}{@{}rcl@{}} Accuracy &=& \frac{TP+TN}{(TP+FP+FN+TN)}\\ Sensitivity (TPR) &=& \frac{TP}{(TP+FN)}\\ Error Rate(FPR) &=& \frac{FP}{(FP+TN)} \end{array} $$

where *TP*, *TN*, *FP* and *FN* refers to true positive, true negative, false positive and false negative respectively. *TP* represents the number of pixels which are part of the region that are labeled correctly by both the method and the ground truth. *TN* represents the number of pixels which are part of the background region and labeled correctly by both the method and the ground truth. *FP* represents the number of pixels labeled as a part of the region by the method but labeled as a part of the background by the ground truth. Finally, *FN* represents the number of pixels labeled as a part of the background by the system but labeled as a part of the region in ground truth. The results of applying the above criteria on the RNFLT are shown in Table 2.

**Table 1:** Performance evaluation with mean and standard deviation (STD) of RMSE and MAD for 7 retinal layers on 150 SD-OCT B-Scan images (Units in pixels)

Retinal layer	Mean MAD (STD)	Mean RMSE (STD)
NFL	0.2688 (0.0185)	0.0165 (0.0121)
GCL+IPL	0.5762 (0.0590)	0.0415 (0.0378)
INL	0.6307 (0.0785)	0.0373 (0.0612)
OPL	0.4839 (0.0410)	0.0446 (0.0335)
ONL+IS	0.6596 (0.0823)	0.0592 (0.0329)
OS	0.4401 (0.0362)	0.0328 (0.0156)
RPE	0.4369 (0.3291)	0.0311 (0.0142)

**Table 2:** Retinal nerve fibre layer thickness (RNFLT) mean accuracy, sensitivity, error rate and dice with their standard deviation (STD) on 150 OCT images

Measure	*Mean*	*STD*
Accuracy	0.9836	0.0370
Sensitivity	0.9692	0.0468
Error Rate	0.0629	0.0743
Dice score	0.9712	0.0541

The use of prior knowledge enables the use of a compromise to replace user input in fully autamated segmentation methods. For example, graph-based methods [5, 7], which rely on the fact retinal layers spread across the image horizontally, which enabled the addition of two columns to either side of the image, such that the cut can traverse within these columns easily. Further, level-set methods [8] and [10] relying on the OCT topology and intensity variation individual layers to constrain the evolution of the level-set functional.

Furthermore, the output of the proposed method (Fig. 4) provides individual layer information that is vital for the diagnosis of eye diseases. Storing these annotated images with the corresponding notes will facilitate the understanding of physician’s rationale for a particular diagnosis. Also, with the variation of individual layer properties, having the images will aid in establishing standards among communities. For example, the average thickness of a layer based on recent evaluations within the community. With such information, standards can be established for eye diagnosis within a particular region (prevalence studies have tried to classify the risk of prevalent eye diseases based on region, age, race and gender [22]). This information, can also aid in the development of robust statistical based model for the segmentation of OCT images.

## Conclusions

Unlike other clinical data that are normally recorded with their inherent and abstract structure, medical images such as the OCT images are usually acquired in the large, raw format. This lack of structured and high-level information in retinal images has limited their potential in clinical practice and healthcare analytics. To address this problem, we have developed a comprehensive and fully automatic method for annotation of retinal layers in OCT images by integrating an advanced method of weight calculation into the graph-cut framework. The introduction of stabilizer enables the method to adapt to intensity inhomogeneity of OCT images in the preprocessing step, while the reassignment of weight aids the method in avoiding wrong paths, which consequently improves the accuracy of the method in identifying actual layer boundaries.

We sequentially segmented 7 layers across 8 boundaries by utilizing prior knowledge of the unique layer characteristics. It is evident that the use of prior knowledge has the potential to improve segmentation algorithms. Having automatic methods that could extract this knowledge will play a vital role on how OCT image analysis evolves. Our method addresses the need for algorithmic frameworks that could be adapted to large applications of OCT images. Integrating images with EHR continuously will be an ideal way to progress towards personalizing health care.
